# PRRX1-OLR1 axis supports CAFs-mediated lung cancer progression and immune suppression

**DOI:** 10.1186/s12935-024-03436-9

**Published:** 2024-07-15

**Authors:** Yunhao Sun, Kaijun Ying, Jian Sun, Yao Wang, Limin Qiu, Mingming Ji, Lin Sun, Jinjin Chen

**Affiliations:** 1 Department of Thoracic Surgery, The First People’s Hospital of Yancheng City, The Yancheng Clinical College of Xuzhou Medical University, Yancheng, 224005 Jiangsu People’s Republic of China; 2 Department of Endocrinology, The First People’s Hospital of Yancheng City, The Yancheng Clinical College of Xuzhou Medical University, Yancheng, 224005 Jiangsu People’s Republic of China; 3 Department of Oncology, The First People’s Hospital of Yancheng City, The Yancheng Clinical College of Xuzhou Medical University, Yancheng, 224005 Jiangsu People’s Republic of China

**Keywords:** Lung cancer, CAFs, PRRX1, OLR1, Immune surveillance

## Abstract

**Objective:**

To investigate the mechanism by which cancer-associated fibroblasts (CAFs) affect the growth and immune evasion of lung cancer cells.

**Methods:**

Initially, datasets comparing CAFs with normal fibroblasts were downloaded from the GEO dataset GSE48397. Genes with the most significant differential expression were selected and validated using clinical data. Subsequently, CAFs were isolated, and the selected genes were knocked down in CAFs. Co-culture experiments were conducted with H1299 or A549 cells to analyze changes in lung cancer cell growth, migration, and immune evasion in vitro and in vivo. To further elucidate the upstream regulatory mechanism, relevant ChIP-seq data were downloaded from the GEO database, and the regulatory relationships were validated through ChIP-qPCR and luciferase reporter assays.

**Results:**

OLR1 was significantly overexpressed in CAFs and strongly correlated with adverse prognosis in lung cancer patients. Knockdown of OLR1 markedly inhibited CAFs’ support for the growth and immune evasion of lung cancer cells in vitro and in vivo. ChIP-seq results demonstrated that PRRX1 can promote OLR1 expression by recruiting H3K27ac and H3K4me3, thereby activating CAFs. Knockdown of PRRX1 significantly inhibited CAFs’ function, while further overexpression of OLR1 restored CAFs’ support for lung cancer cell growth, migration, and immune evasion.

**Conclusion:**

PRRX1 promotes OLR1 expression by recruiting H3K27ac and H3K4me3, activating CAFs, and thereby promoting the growth, migration, and immune evasion of lung cancer cells.

**Supplementary Information:**

The online version contains supplementary material available at 10.1186/s12935-024-03436-9.

## Highlights

1. OLR1 is significantly overexpressed in lung cancer-associated fibroblasts and is associated with adverse patient outcomes.
2. OLR1^+^ CAFs promote the growth of lung cancer cells in vitro and in vivo, as well as immune evasion.
3. PRRX1 serves as the master transcription factor regulating OLR1^+^ CAFs.
4. PRRX1 can recruit super enhancer modifications in the cis-elements of the OLR1 gene.
5. Overexpression of OLR1 weakens the inhibitory effect of shPRRX1 on CAFs’ function.

**Supplementary Information:**

The online version contains supplementary material available at 10.1186/s12935-024-03436-9.

## Introduction

Lung cancer represents the second most common (11.4%) and the leading cause of cancer death (18%) worldwide in 2020 [1]. Approximately 85% of the diagnosed patients have a class of histological subtypes recognized as non-small cell lung cancer (NSCLC), with lung adenocarcinoma (LUAD) and lung squamous cell carcinoma (LUSC) representing the most frequent subtypes [2]. Clinically, only a small portion of patients (about 20–25%) present with early-stage NSCLC (stage I or II) at diagnosis and undergo surgical resection, whereas a larger portion present with advanced disease mainly underwent conventional chemotherapy and radiation therapy, targeted therapy, and immunotherapy [3, 4]. However, the immune checkpoint molecules such as programmed cell death protein 1 and its ligand programmed death-ligand 1 (PD-L1) expressed by tumor, immune, and stromal cells suppress CD8^+^ T cells-mediated cytotoxic immune response and lead to immunosuppression [5–7].

In recent years, the tumor microenvironment (TME) has received increasing attention due to its crucial roles in tumor immune suppression, distant metastasis, local resistance and the targeted therapy response [8–11]. The TME is a highly complicated system mainly composed of tumor cells, infiltrating immune cells (such as macrophages, dendritic cells and lymphocytes), cancer-associated stromal cells [such as cancer-associated fibroblasts (CAFs)], endothelial cells and lipocytes, along with the extracellular matrix (ECM) and multiple signaling molecules [12, 13]. As one of the most important stromal components in the TME, CAFs have shown biological heterogeneity in many aspects, including the cell of origin, phenotype and function [14, 15]. Originating from a variety of cell types, CAFs are characterized by increased expression of markers such as alpha smooth muscle actin (α-SMA), fibroblast activation protein (FAP), fibroblast-specific protein 1 (FSP1), platelet-derived growth factor receptor (PDGFR)-α/β and vimentin [16]. Most of CAF subpopulations usually exhibit cancer-promoting effects, while the discovery of cancer-restraining CAFs (rCAFs), which are reported to exert inhibitory effects on tumor progression, indicates that some subsets are just the opposite [17]. Substantial previous reports have demonstrated that CAFs participate in multiple stages of tumor development through diverse pathways [18–20]. Through bidirectional signaling with tumor cells and other cells mediated by CAF-derived cytokines, chemokines, growth factors and exosomes within the TME, CAFs not only facilitate tumor proliferation but also induce immune evasion of cancer cells [14, 21, 22]. In lung cancer, the abundance of CAFs in TME has been associated with the exclusion of T cells and immune suppression [23]. However, the roles of CAFs in the growth and development of lung cancer cells, in immune evasion, as well as the regulatory mechanisms remain largely unknown. This study was therefore performed to identify key molecular mechanisms involved in the immunosuppressive and tumor promoting roles of CAFs in lung cancer.

## Material and methods

### Bioinformatics analysis

We downloaded the dataset GSE48397 from the GEO database, which includes 5 samples of normal lung fibroblasts and 5 samples of CAFs. The data were corrected and normalized using the Limma package, and a heatmap was generated using the ggplot2 package. Additionally, ChIP-seq data for PRRX1 in CAFs were downloaded from the GEO database. MACS2 was employed to analyze PRRX1’s ChIP-seq data, and call4peaking was used to assess the binding of PRRX1 to the CAF genome.

### Clinical sample collection

We collected tumor tissue samples from 48 cases of lung cancer from January 2019 to June 2022 in the oncology and respiratory departments of our hospital. These samples were routinely dehydrated and embedded in paraffin for immunohistochemistry (IHC) analysis. Clinical information for all enrolled patients was obtained from our hospital's Health Information System (HIS), including gender, age, staging, pathological features, smoking status, treatment plans, clinical outcomes, and more [24].

### Inclusion criteria

1. Patients aged 18 years and older.
2. Confirmed diagnosis of lung adenocarcinoma through histopathological examination.
3. Staged as IV according to the AJCC Eighth Edition Lung Cancer TNM Staging.
4. Provision of approved histopathological samples.
5. Met one or more of the following criteria: Eastern Cooperative Oncology Group (ECOG) PS score of 0 or 1.
6. Sufficient tumor tissue available for PD-L1 expression analysis.
7. At least one record of PD-1/PD-L1 treatment.

### Exclusion criteria

1. Patients with sensitive gene mutations (EGFR, ALK, ROS1).
2. Patients who have already received immune checkpoint inhibitor therapy (e.g., PD-1, PD-L1, CTLA-4, etc.).
3. Patients with known autoimmune diseases, such as systemic lupus erythematosus, rheumatoid arthritis, scleroderma, etc.
4. Currently undergoing treatment with other experimental drugs.
5. Patients with severe liver, kidney, or heart diseases.
6. Other situations that do not meet the study requirements.

Clinical Treatment Response Evaluation: Clinical treatment response was assessed using modified Response Evaluation Criteria in Solid Tumors (mRECIST) criteria, categorized as follows: Complete Remission (CR), Partial Remission (PR),Stable Disease (SD), Progressive Disease (PD). Progression-Free Survival (PFS): PFS is defined as the time from the start of treatment to disease progression or death from any cause. The first treatment response evaluation was conducted after two cycles of treatment, with subsequent evaluations performed every two treatment cycles. Patients who achieved CR, PR, or SD for at least 4 weeks after receiving immunotherapy were classified as responders, while patients with disease progression were classified as non-responders.

### Immunohistochemistry

Paraffin-embedded sections of lung adenocarcinoma tissue were used for immunohistochemical staining. The sections underwent deparaffinization in xylene, followed by gradual ethanol dehydration. They were then rinsed three times in PBS for 5 min each time. To block endogenous peroxidase activity, the sections were immersed in a 3% hydrogen peroxide solution. After another rinse with PBS, antigen retrieval was performed in 98 °C citrate solution for 30–60 s, followed by PBS washing. Primary antibodies, including OLR1 (ab232837, 1:200), PRRX1 (ab20034, 1:500), and PD-L1, were applied and incubated overnight at 4 °C. After PBS washing, secondary antibodies were added, and the sections were incubated at room temperature for 40 min. Following another PBS wash, DAB staining was performed. Counterstaining was done with hematoxylin, and sections were dehydrated in a graded ethanol series, cleared in xylene, and mounted with neutral resin for observation under an optical microscope. Data collection was based on the percentage of stained cells and staining intensity. OLR1 was considered positive when localized in the cytoplasm of tumor cells, occasionally with positive membrane expression. Positivity was determined by the presence of brownish-yellow granules in the cytoplasm and/or cell membrane. PRRX1 was considered positive when nuclear staining exhibited yellow or light brown granules. For each slide, five high-power fields with a higher number of stained cells were chosen, and the absolute quantity of PRRX1-positive cells in the tumor stroma was determined by averaging cell counts. Protein expression of OLR1 or PRRX1 was evaluated using the H-score system. “Pi” represents the percentage of cells at a particular intensity level “i,” with values ranging from 0 to 100%. “I” represents the staining intensity, with values of 0 (no staining evidence), 1 (weak staining), 2 (moderate staining), and 3 (strong staining). The final H-score was calculated by summing the products of “I” and “Pi” (H-score = (0P0) + (1P1) + (2P2) + (3P3)), with a range of scores from 0 to 300.

### Isolation and extraction of CAFs

CAFs were isolated from fresh LUAD tissues. After surgical resection, samples were collected in tissue storage buffer (Miltenyi) and washed in phosphate-buffered saline (PBS) containing 1% antibiotics-antimycotics (Gibco, Life Technologies). Tissues were then minced into small pieces (1–2 mm) and digested using the Human Tumor Dissociation Kit and gentleMACS Octo Dissociator following the manufacturer's instructions. The digested samples were sequentially filtered through a 70-micron cell strainer. Cells were collected by centrifugation at 250 g for 5 min and cultured in Dulbecco’s Modified Eagle Medium (DMEM) (Gibco, Carlsbad, CA, USA) supplemented with 10% fetal bovine serum (FBS) and 1% antibiotics-antimycotics at 37 °C. The culture medium was changed every 3 days. Primary CAFs were characterized as negative for EpCAM, CD45, and CD31, and positive for FAP and α-SMA. To knock down or overexpress specific genes, primary fibroblasts were transduced with lentiviral vectors [multiplicity of infection (MOI) of 100] at 37 °C with 5 μg mL^−1^ polybrene (Sigma). The targeting sequences of each shRNA are provided in Table 1.

**Table 1:** shRNA sequences

Symbol	ds Oligos
ShOLR1-#1	5ʹ-CACCGGGCAGCTTTAACTCTGAATCCGAAGATTCAGAGTTAAAGCTGCCC-3ʹ
ShOLR1-#2	5ʹ-CACCGGCAGCTTTAACTCTGAATCCCGAAGGATTCAGAGTTAAAGCTGCC-3ʹ
shPRRX1-#1	5ʹ-CACCGCGTTTGGTGTTGATTCGAGCCGAAGCTCGAATCAACACCAAACGC-3ʹ
shPRRX1-#2	5ʹ-CACCGCGTTTGGTGTTGATTCGAGCGAGAGCTCGAATCAACACCAAACGC-3ʹ

### Immunofluorescence

Cells were plated on sterile glass slides and cultured for 48 h. Subsequently, cells were fixed with 4% paraformaldehyde for 15 min, permeabilized with 0.25% Triton-X 100 for 10 min, and then blocked with 3% BSA for 1 h. Immunofluorescence staining was performed using antibodies against Vimentin, α-SMA, FSP1, F-actin, and Fibronectin (dilution 1:100, Abcam). After washing with phosphate-buffered saline (PBS), cells were incubated with Alexa Fluor 488 or Alexa Fluor 546 secondary antibodies (Life Technologies). Cell nuclei were stained with DAPI, and observations were made using a FV-1000 laser scanning confocal microscope. Quantitative analysis was performed on cells from at least 300 cells in three independent experiments.

### qPCR

RNA was extracted from CAFs or LC cells using the RNAeasyTM Plus Animal RNA Isolation Kit with Spin Column (Beyotime, Jiangsu, China). cDNA synthesis was performed using SuperScript III (Takara, Dalian, China) following the manufacturer's protocol. Real-time PCR analysis was carried out using the Applied Biosystems 7500 Real-Time PCR System, as per the manufacturer's instructions. Reactions were performed for three independent experiments, and the results were normalized to β-actin. The primer sequences used can be found in Table 2. The mean ± SD of three independent experiments is presented.

**Table 2:** qPCR primers

Symbol	Fwd	Rvs
OLR1	GAAACCCTTGCTCGGAAGCTGA	CAGATCCAGTCTTGCGGACAAG
PRRX1	GACCAACCGATTATCTCTCCTGG	CAGTCTCAGGTTGGCAATGCTG
PDL1	TGCCGACTACAAGCGAATTACTG	CTGCTTGTCCAGATGACTTCGG
GAL9	CCAAGTCCATCATCCTGTCAGG	GGTGTTACGGACCACAGCATTC
TIM3	GACTCTAGCAGACAGTGGGATC	GGTGGTAAGCATCCTTGGAAAGG
GAPDH	GTCTCCTCTGACTTCAACAGCG	ACCACCCTGTTGCTGTAGCCAA

### Western blot

Total protein was extracted using cold radioimmunoprecipitation assay (RIPA) buffer containing protease and phosphatase inhibitors. Cell lysates were separated by 8–10% SDS-PAGE and transferred to polyvinylidene fluoride (PVDF) membranes. Rabbit or mouse IgG antibodies conjugated with horseradish peroxidase (Santa Cruz) were used as secondary antibodies. Protein bands labeled on the membranes with antibodies were detected using the G-BOX iChemi XT instrument (Syngene).

### Co-culture

Co-culture experiments were established using Transwell membranes with a pore size of 0.4 µm (Merck Millipore, USA) in 12-well plates. Co-culture was conducted for 3–6 days, with LC cells (5 × 10^4^ cells) seeded on the permeable membrane, and CAFs cells (initially 5 × 10^4^ cells) prepared for further cell-based experiments beneath the membrane.

### CCK-8 assay

Cell viability was assessed using the Cell Counting Kit-8 (CCK-8) assay kit (Sigma) following the manufacturer’s instructions. LC cells were digested with trypsin, counted, and then seeded in 96-well plates at a density of 2 × 10^3^ cells per well. Cells were cultured at 37 °C. Adherent cells were incubated with CCK-8 dilution for 1 h, followed by measuring the absorbance at a wavelength of 450 nm for each well.

### SA-β-gal assay

Senescence-associated β-Galactosidase (SA-β-Gal) activity was measured using the senescence-associated β-Galactosidase staining kit (Beyotime, Shanghai, China) according to the manufacturer's instructions. Briefly, cells grown in 6-well plates were first washed and fixed for 15 min at room temperature, then incubated overnight at 37 °C with SA-β-gal working solution. For xenograft tumor tissues, frozen tissues were cut into 4 μm sections using a cryostat and mounted on glass slides with a positive charge. The sections were then fixed and stained as described above. SA-β-Gal-positive cells, appearing blue (light or dark blue), were determined as the percentage of stained cells from five random fields relative to the total cell count.

### Matrigel invasion assay

The Matrigel invasion assay was performed in 24-well Transwell culture plates. In brief, 40 μL of Matrigel (1 mg/mL, BD) was coated on 8 μm polycarbonate membrane filters. Approximately 3 × 10^4^ H1299 or A549 cells were re-suspended and then seeded in the upper chambers containing FBS-free culture medium and the lower chambers containing complete growth medium with 10% FBS, respectively. Cells were incubated at 37 °C for 24 or 48 h. Non-invasive cells on the upper side of the invasion membrane were removed, and cells on the lower surface were stained with hematoxylin. The average number of cells in each field was determined by counting cells in six random fields per well. Cells were counted in four different fields from three independent experiments.

### ChIP-qPCR

ChIP experiments were performed using the EZ-Magna ChIP kit (Millipore, Billerica, MA, USA). Approximately 5 × 10^6^ cells were fixed with 1% formaldehyde (final concentration) at room temperature for 10 min. Fixation was stopped by adding 1/10 volume of 1.25 M glycine and incubating at room temperature for 5 min. The sonication step was carried out in a Covaris sonicator (5 min, 20 s on, 20 s off) with 200 µg of protein-chromatin complexes used for each immunoprecipitation. Antibody-protein complexes were captured using pre-blocked dynabeads protein G (Invitrogen). qPCR analysis was performed using SYBR Green (Takara) on an ABI-7500 instrument (Applied Biosystems), with primers specified in Table 3. The antibodies used included H3K27ac, H3K4me3, PRRX1 (CST), and normal mouse IgG (Millipore).

**Table 3:** ChIP-qPCR primers

Symbol	Fwd	Rvs
OLR1 promoter-#1	AAGTGCTCTCTGCTGCACG	TGGTAGGAGGAAGGAGAGGC
OLR1 promoter-#2	GCAATCCACCCTGGGTAACA	GTCAGGAGTTCGAGACCAGC

### Dual-luciferase reporter assay

Transcriptional activity was analyzed using the dual-luciferase reporter gene assay according to the manufacturer’s instructions (Promega Corporation, Madison, WI, USA). Reporter gene expression was measured and quantified using the Dual-Luciferase Reporter Assay kit (Promega). Luciferase activity was normalized to Renilla luciferase control activity.

### Animal experiments

For in vivo metastasis experiments, each experimental group included 6–8 male C57BL/6 J mice aged 6 weeks. In brief, H1299 cells were mixed with CAFs cells stably expressing low levels of OLR1 in a 1:1 ratio, suspended in 40 μL of serum-free DMEM, and then injected into the tail veins of mice. Starting from the second week, mice were treated with anti-PD-1 therapy (50 mg/kg/week) for 8 weeks, after which the mice were euthanized. Tumor nodules formed on the lung surface were macroscopically identified and counted. Lung tissues were then excised and embedded in paraffin for HE staining to analyze the number of lung nodules, and flow cytometry was used to assess the proportion of immune cell infiltration in the nodules.

### ELISA

Analysis was performed using mouse IL-6, IL-8, IFNg, CSF1 (Absion), and TNF-alpha, TGF-β quantikine ELISA kits (Novus, China) according to the manufacturer’s instructions. In brief, 50 μL of samples and standards were added to wells of a microplate. Then, 100 μL of assay reagent was added to each assay well. After incubation for 20 min, absorbance at 450 nm was measured using an ELISA microplate reader.

### Statistical analysis

Data analysis was performed using SPSS 21.0 (SPSS, Inc., Chicago, IL, USA) statistical software. The data were assessed for normality using the Kolmogorov–Smirnov test, and as the data followed a normal distribution, results are presented as mean ± standard deviation. Comparisons between two groups were conducted using the t-test, while comparisons among multiple groups were analyzed using One-Way ANOVA (analysis of variance). Post hoc pairwise comparisons following ANOVA were conducted using Tukey's multiple comparisons test. Survival curves were generated using the Kaplan–Meier method, and statistical significance was assessed using the Log-rank test. All tests were two-tailed, and a p-value less than 0.05 was considered statistically significant.

## Results

### OLR1 is significantly overexpressed in lung cancer-associated fibroblasts and is associated with poor patient prognosis

**Fig. 1:**
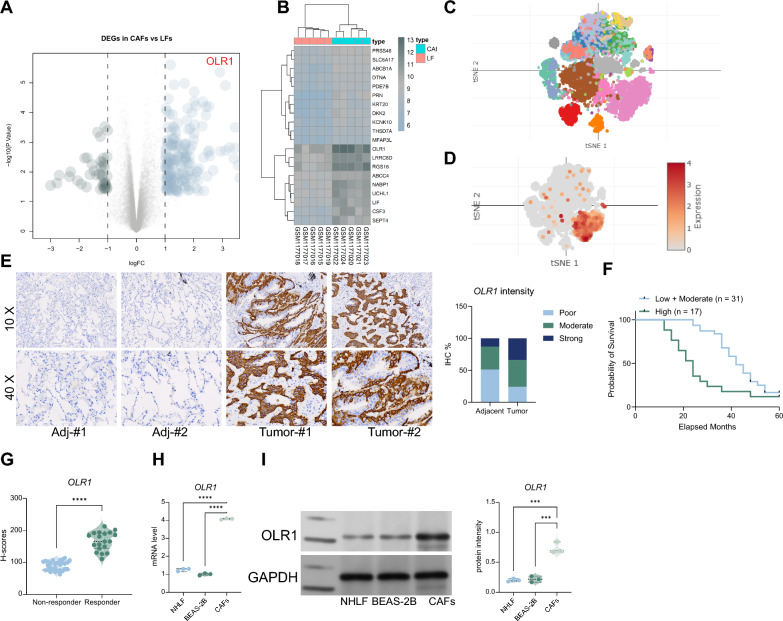
OLR1 is markedly upregulated in lung cancer-associated fibroblasts and is associated with adverse patient outcomes. **A** Differential gene expression analysis of the dataset GSE48397, showing significant overexpression of OLR1 in CAFs compared to normal lung fibroblasts. **B** Volcano plot or heatmap representation of differential gene expression in GSE48397, highlighting OLR1 overexpression in CAFs. **C** UMAP clustering of single-cell RNA-seq data from the dataset GSE240001, showing dimensionality reduction and clustering of lung cancer tumor cells. **D** Expression of CAF-specific markers SDF1 and alpha-SMA in UMAP clusters, indicating specific expression of OLR1 in CAFs. **E** Immunohistochemistry (IHC) analysis of OLR1 staining intensity in tumor tissues from 48 lung cancer patients, showing higher OLR1 expression correlates with worse survival prognosis. **F** Kaplan–Meier survival curve of lung cancer patients, stratified by high and low OLR1 staining intensity, showing poorer survival in patients with high OLR1 expression. **G** Analysis of response to anti-PD-1 therapy in lung cancer patients, showing reduced response in patients with high OLR1 staining intensity. **H**–**I** Quantitative polymerase chain reaction (qPCR) and Western blot (WB) analyses revealing OLR1 mRNA and protein expression levels in cancer-associated fibroblasts (CAFs), normal human lung fibroblasts (NHLF), and bronchial epithelial cells (BEAS-2B). The experiments were repeated three times, and data are presented as mean ± standard deviation. Statistical analysis was performed using one-way ANOVA or 2-Way ANOVA, followed by Tukey’s post-hoc validation, **P < 0.01, ***P < 0.001

We downloaded the dataset GSE48397 from the GEO database, which included 5 samples of normal lung fibroblasts and 5 samples of cancer-associated fibroblasts (CAFs). Differential gene expression analysis revealed that OLR1 was significantly overexpressed in CAFs (Fig. 1A, B). To further confirm the high expression of OLR1 in lung cancer CAFs, we obtained a single-cell dataset of lung cancer tumors, GSE240001, from the GEO database. We first performed dimensionality reduction using the SEURAT package and clustered the data based on UMAP. By selecting CAF-specific markers SDF1 and alpha-SMA, we observed specific expression of OLR1 in CAFs (Fig. 1C, D). Next, we collected tumor tissues from 48 lung cancer patients along with corresponding adjacent non-cancerous tissues. We analyzed the relationship between OLR1 staining intensity and patient prognosis through immunohistochemistry (IHC). We observed that patients with higher OLR1 staining intensity had worse survival prognosis and responded less to anti-PD-1 therapy (Fig. 1E–G). Therefore, we isolated CAFs from lung cancer tissues using enzymatic digestion, and immunofluorescence analysis confirmed the positive expression of CAF markers Vimentin, FSP1, and alpha-SMA (Fig S1A). Furthermore, we found that the expression level of OLR1 in CAFs was significantly higher than that in the NHLF and BEAS-2B cell lines (Fig. 1H, I).

### OLR1^+^ CAFs promote the malignant biological behavior of lung cancer cells and immune evasion

**Fig. 2:**
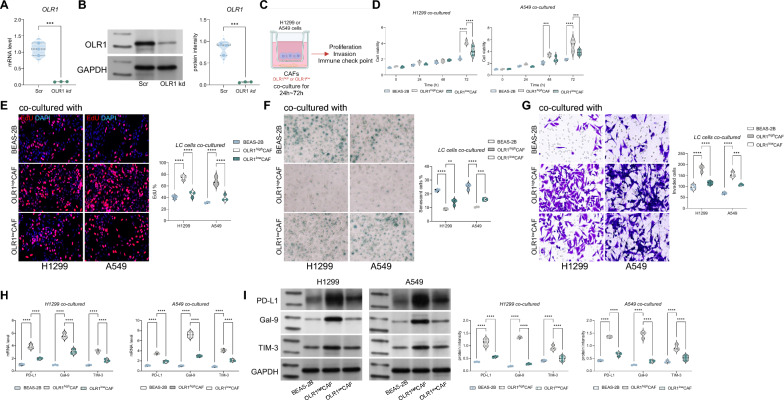
Promotion of malignant biological behaviors and immune escape of lung cancer cells by OLR1^+^ CAFs. **A** Schematic representation of the experimental design. Knockdown of OLR1 in CAFs and establishment of co-culture systems with BEAS-2B, CAFs, and H1299 or A549 lung cancer cells. **B** Confirmation of OLR1 knockdown in CAFs by qRT-PCR or Western blot analysis. **C** Schematic diagram showing the co-culture setup with BEAS-2B, CAFs, and lung cancer cells (H1299 or A549). **D** Proliferation assay of H1299 and A549 cells co-cultured with OLR1high or OLR1low CAFs, showing significantly increased proliferation in the presence of OLR1high CAFs. **E** DNA replication assay (e.g., EdU incorporation assay) demonstrating enhanced DNA replication in H1299 and A549 cells co-cultured with OLR1high CAFs. **F** Senescence assay of H1299 and A549 cells, showing reduced senescence levels when co-cultured with OLR1high CAFs. **G** Transwell invasion assay of H1299 and A549 cells, indicating significantly promoted invasion in the presence of OLR1high CAFs. **H** Western blot or flow cytometry analysis of immune evasion-related markers (PD-L1, Galectin-9, and TIM-3) in H1299 and A549 cells, showing increased expression levels when co-cultured with OLR1high CAFs. **I** Comparative analysis of immune evasion-related markers in H1299 and A549 cells co-cultured with OLR1low CAFs, showing reduced expression levels compared to co-culture with OLR1high CAFs. Experiments were performed in triplicate, and data are presented as mean ± standard deviation. Statistical analysis was conducted using one-way ANOVA or 2-Way ANOVA, followed by Tukey’s post-hoc validation, **P < 0.01, ***P < 0.001

To elucidate the effect of OLR1^+^ CAFs on the growth of lung cancer cells, we first knocked down the expression of OLR1 in CAFs and established a co-culture system of BEAS-2B, CAFs, with H1299 or A549 cells (Fig. 2A–C). We found that the proliferation or DNA replication ability of H1299 or A549 cells was significantly promoted after co-culturing with OLR1^high^ CAFs (Fig. 2D, E). Furthermore, we observed that OLR1^high^ CAFs could attenuate the senescence level of H1299 or A549 cells (Fig. 2F). Surprisingly, in the Transwell system, OLR1^high^ CAFs significantly promoted the invasion of H1299 and A549 cells (Fig. 2G). Previous studies have demonstrated that cytokines secreted by CAFs can promote immune evasion of lung cancer cells. Therefore, we examined the expression of immune evasion-related markers in H1299 and A549 cells. We found that OLR1^high^ CAFs significantly increased the expression levels of PD-L1, Galectin-9, and TIM-3 (Fig. 2H, I). Contrary to our expectations, when co-cultured with CAFs with knocked down OLR1 (OLR1^low^), the malignant biological behavior of H1299 and A549 cells and the expression of immune evasion-related markers were significantly reduced (Fig. 2D–I). These results indicate that OLR1 is a key gene that affects the support of CAFs for the growth and immune evasion of lung cancer cells.

Based on the above results, it can be inferred that co-culturing with cancer-associated fibroblasts (CAFs) significantly promotes the malignant biological behavior of lung cancer cells H1299 or A549. However, knocking down OLR1 in CAFs attenuates the promoting effect of CAFs on the malignant biological behavior of lung cancer cells.

### OLR1^+^ CAFs promote the in vivo growth of lung cancer cells and resistance to anti-PD-1 therapy

**Fig. 3:**
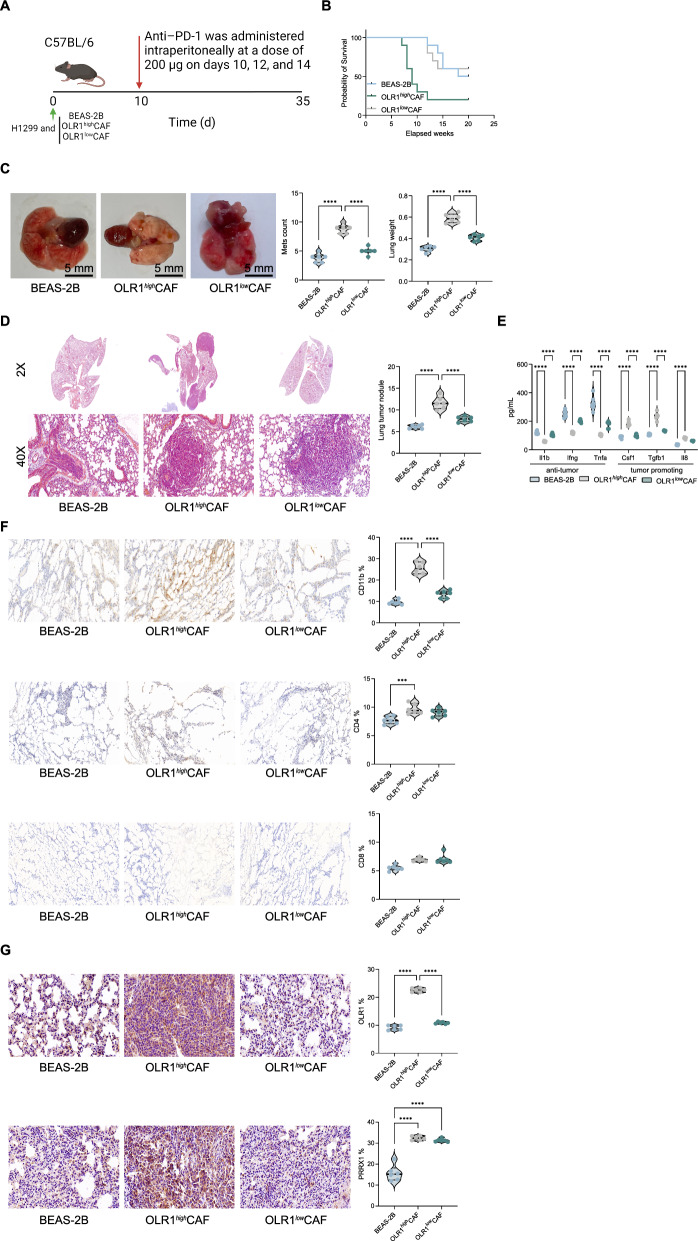
Promotion of in vivo growth of lung cancer cells and resistance to anti-PD-1 therapy by OLR1^+^ CAFs. **A** Schematic representation of the experimental design. H1299 cells were mixed with BEAS-2B, OLR1high, or OLR1low CAFs in a 1:1 ratio and injected into C57 mice via the tail vein, followed by anti-PD-1 therapy. **B** Response to anti-PD-1 therapy in mice inoculated with BEAS-2B/H1299, OLR1high/H1299, and OLR1low/H1299 cells, showing the highest response in the BEAS-2B/H1299 group and the lowest response in the OLR1high/H1299 group. **C** Analysis of metastatic nodules in lung tissues of mice, demonstrating that CAFs promote the formation of metastatic foci of H1299 cells in the lung, with reduced metastasis observed in the OLR1low group. **D** Quantification of the number of metastatic nodules in lung tissues, showing a significant increase in nodules in the OLR1high group compared to the OLR1low group. **E** Measurement of tumor-promoting cytokine levels in mouse tissues. Tumor-promoting cytokine levels are significantly increased in mice inoculated with CAFs, with attenuation observed in the OLR1low group. **F** Flow cytometry analysis of immune cell infiltration in lung tumor nodules. OLR1high CAFs attract more tumor-associated macrophages (TAMs), while the numbers of CD4 and CD8 cells show no significant difference. **G** Immunohistochemistry analysis of OLR1 staining intensity in lung metastatic nodules, showing higher intensity in nodules from mice inoculated with OLR1high CAFs compared to OLR1low CAFs. **H** Immunohistochemistry analysis of PRRX1 staining intensity in lung metastatic nodules, showing higher intensity in nodules from mice inoculated with OLR1high CAFs compared to OLR1low CAFs. Each group consisted of 6–8 mice, and data are presented as mean ± standard deviation. Statistical analysis was performed using one-way ANOVA or 2-Way ANOVA, followed by Tukey’s post-hoc validation, **P < 0.01, ***P < 0.001

To further confirm the effect of CAFs and OLR1 on the in vivo growth and metastasis of lung cancer cells, we mixed H1299 cells with BEAS-2B, OLR1high, and OLR1low CAFs in a 1:1 ratio and injected them into C57 mice via the tail vein, followed by anti-PD-1 therapy (Fig. 3A). We found that mice inoculated with BEAS-2B/H1299 cells had the highest response to anti-PD-1, followed by OLR1low/H1299, while mice inoculated with OLR1high showed the lowest response to anti-PD-1 therapy (Fig. 3B). Subsequently, we analyzed the number of metastatic nodules in the lung tissues of the mice. We observed that CAFs significantly promoted the formation of metastatic foci of H1299 in lung tissues, but reducing the expression of OLR1 in CAFs significantly weakened their support for H1299 cell metastasis in vivo (Fig. 3C, D). Furthermore, to analyze the impact of CAFs on the immune microenvironment, we measured the levels of tumor-promoting cytokines and anti-tumor cytokines in the mouse tissues. We observed that the levels of tumor-promoting cytokines were significantly increased in the mice inoculated with CAFs, but knocking down OLR1 attenuated this effect (Fig. 3E). Additionally, we further analyzed the infiltration ratio of various immune cells in the lung tumor nodules formed using flow cytometry. OLR1high CAFs attracted more TAM infiltration, while the numbers of CD4 and CD8 cells showed no significant difference (Fig. 3F). Additionally, we confirmed through immunohistochemistry that the staining intensity of both OLR1 and PRRX1 was significantly higher in lung metastatic nodules of mice inoculated with OLR1-high CAFs compared to those inoculated with OLR1-low CAFs (Fig. 3G, H). These results confirm that OLR1high CAFs enhance the in vivo growth, metastasis, and immune evasion of lung cancer cells. The high expression of OLR1 in CAFs promotes the formation of metastatic nodules, increases tumor-promoting cytokine levels, and attracts more TAM infiltration. Conversely, reducing OLR1 expression in CAFs weakens these effects, demonstrating the critical role of OLR1 in the supportive function of CAFs for lung cancer progression.

### PRRX1 as a master transcription factor regulating OLR1^+^ CAFs

**Fig. 4:**
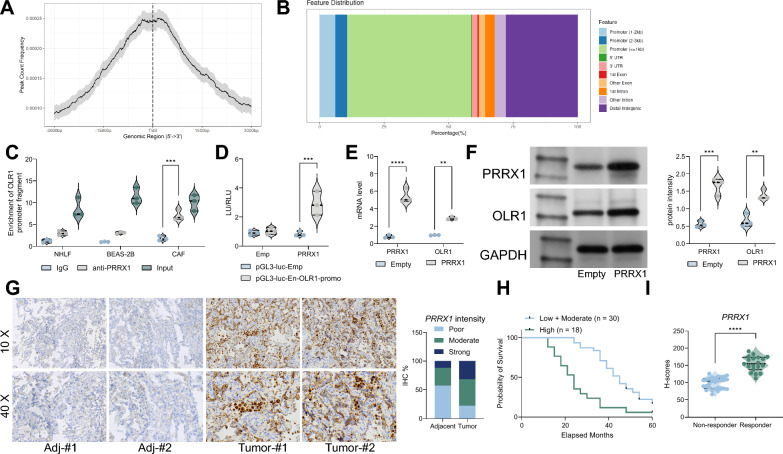
PRRX1 as the master transcription factor regulating OLR1^+^ CAFs. **A** Distribution of PRRX1 binding sites from ChIP-seq data in CAFs, showing enrichment mainly in the CDS region. **B** Enrichment of PRRX1 near the upstream elements of OLR1 in CAFs based on ChIP-seq analysis. **C** ChIP-qPCR analysis of PRRX1 binding to the OLR1 promoter in BEAS-2B, NHLF, and CAFs, demonstrating significantly higher binding in CAFs. **D** Schematic representation of the luciferase reporter assay using the OLR1 promoter (pGL3-luc-En-OLR1-promo) transfected into HEK293T cells along with a PRRX1 overexpression plasmid. **E** Luciferase activity assay results showing that PRRX1 overexpression significantly increases OLR1 promoter activity in HEK293T cells. **F** Western blot or qRT-PCR analysis of OLR1 expression levels in CAFs overexpressing PRRX1, demonstrating increased OLR1 expression. **G** Immunofluorescence or immunohistochemistry analysis confirming increased OLR1 expression in CAFs overexpressing PRRX1. **H** Analysis of PRRX1 expression levels in tumor tissues from 48 lung cancer patients via immunohistochemistry or qRT-PCR. **I** Kaplan–Meier survival curve based on PRRX1 expression levels in lung cancer patients, showing poorer prognosis in patients with higher PRRX1 expression. **J** Analysis of response to anti-PD-1 therapy based on PRRX1 expression levels in lung cancer patients, demonstrating reduced response in patients with higher PRRX1 expression. Experiments were repeated three times, and data are presented as mean ± standard deviation. Statistical analysis was performed using one-way ANOVA or 2-Way ANOVA, followed by Tukey’s post-hoc validation, **P < 0.01, ***P < 0.001

In previous studies, it was suggested that PRRX1 is a master transcription factor of myofibroblastic cancer-associated fibroblasts [25]. However, there has been no in-depth investigation into the downstream effects of PRRX1. Therefore, we hypothesized whether PRRX1 could regulate OLR1 expression through transcriptional control and promote CAF activation in lung cancer. First, we downloaded ChIP-seq data for PRRX1 in CAFs from the GEO database and found that PRRX1 is mainly distributed in the CDS region (Fig. 4A). Furthermore, we observed a significant enrichment of PRRX1 in the upstream elements of OLR1 in CAFs (Fig. 4B). To further analyze the regulation of OLR1 by PRRX1, we performed ChIP experiments using anti-PRRX1 antibodies to investigate the binding of PRRX1 to the OLR1 promoter in BEAS-2B, NHLF, and CAFs. The results showed that PRRX1 binding near the promoter was significantly higher in CAFs than in BEAS-2B or NHLF (Fig. 4C). To validate our experimental results, we transfected HEK293T cells with a luciferase reporter vector containing the OLR1 promoter (pGL3-luc-En-OLR1-promo) along with an overexpression plasmid for PRRX1. We found that PRRX1 significantly increased luciferase activity in the cells (Fig. 4D, E). Furthermore, to confirm the regulatory role of PRRX1 on OLR1 in CAFs, we overexpressed PRRX1 in CAFs and observed an increase in OLR1 expression levels (Fig. 4F, G). In a clinical context, we found that the expression level of PRRX1 in tumor tissues from 48 lung cancer patients was positively correlated with poor prognosis and a reduced response to anti-PD-1 therapy (Fig. 4H–J).

### PRRX1 recruits super enhancer modifications in OLR1 gene cis-elements

**Fig. 5:**
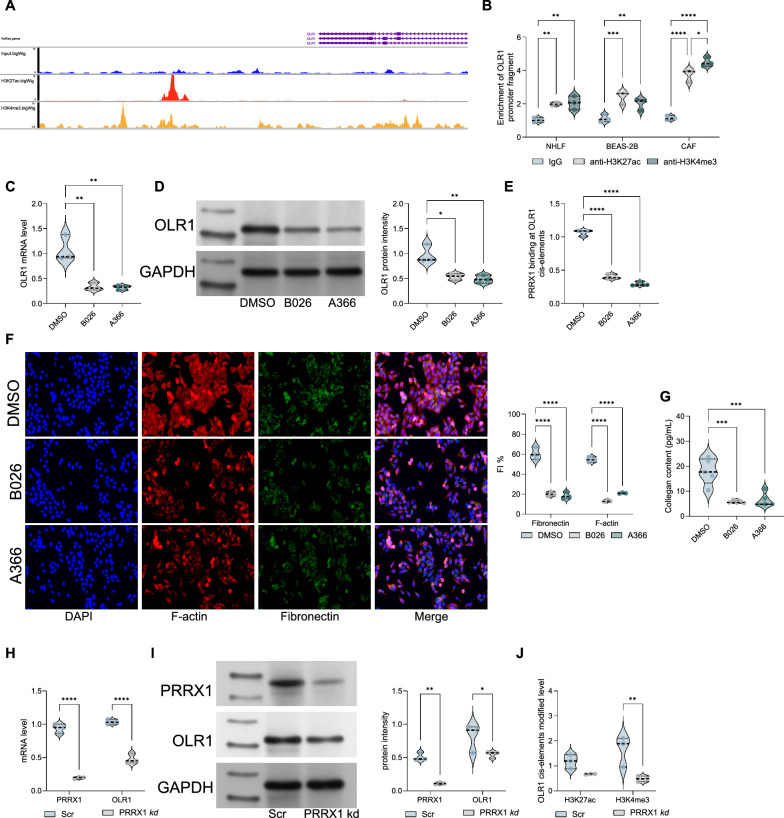
PRRX1 can recruit super enhancer modifications in OLR1 gene cis-elements. **A** Analysis of ChIP-seq data showing dense H3K27ac and H3K4me3 modifications near the upstream 5–10 kb region of the OLR1 CDS, indicating super enhancer signatures. **B** ChIP-qPCR analysis using anti-H3K27ac or H3K4me3 antibodies, demonstrating enrichment of OLR1 cis-elements in CAFs. **C** Effects of the H3K27ac inhibitor B026 and the H3K4me3 inhibitor A-366 on OLR1 mRNA and protein expression levels in CAFs. **D** Western blot or qRT-PCR analysis showing reduced OLR1 protein expression in CAFs treated with B026 or A-366 inhibitors. **E** ChIP-qPCR analysis of PRRX1 binding to the OLR1 promoter in CAFs treated with B026 or A-366 inhibitors, showing reduced binding. **F** Expression levels of Fibronectin and F-actin in CAFs treated with B026 or A-366 inhibitors, indicating reduced activation phenotype. **G** Collagen content assay in CAFs treated with B026 or A-366 inhibitors, showing reduced collagen production. **H** ChIP-qPCR analysis showing reduced enrichment of H3K27ac upstream of OLR1 in CAFs with PRRX1 knockdown. **I** ChIP-qPCR analysis showing reduced enrichment of H3K4me3 upstream of OLR1 in CAFs with PRRX1 knockdown. Experiments were repeated three times, and data are presented as mean ± standard deviation. Statistical analysis was performed using one-way ANOVA or 2-Way ANOVA, followed by Tukey’s post-hoc validation, **P < 0.01, ***P < 0.001

In our further investigation, PRRX1 was found to promote the enrichment of super enhancer signatures H3K27ac and H3K4me3. We downloaded ChIP-seq data for H3K27ac and H3K4me3 super enhancer signatures and observed dense H3K27ac and H3K4me3 modifications near the upstream 5–10 kb region of the OLR1 CDS (Fig. 5A). Similarly, we performed ChIP experiments using anti-H3K4me3 or H3K27ac antibodies and found that more OLR1 cis-elements were enriched in CAFs (Fig. 5B). To confirm the role of H3K27ac and H3K4me3 in CAF activation and OLR1 regulation, we treated CAFs with the broad-spectrum H3K27ac inhibitor B026 and the H3K4me3 inhibitor A-366. We observed a significant reduction in OLR1 mRNA and protein expression levels (Fig. 5C, D). Additionally, the binding of PRRX1 to the OLR1 promoter was significantly reduced (Fig. 5E). We also found that after treatment with B026 or A366, the expression levels of Fibronectin and F-actin in CAFs were significantly reduced (Fig. 5F), and the collagen content produced was also significantly reduced (Fig. 5G). Furthermore, when PRRX1 was knocked down in CAFs, we observed a significant reduction in the enrichment of H3K27ac and H3K4me3 upstream of OLR1 (Fig. 5H, I). These results suggest that PRRX1 can regulate OLR1 by recruiting H3K27ac and H3K4me3 modifications, thereby promoting CAF activation.

### Knocking down PRRX1 suppresses the appearance of CAF-related phenotypes and their support for lung cancer cell growth

**Fig. 6:**
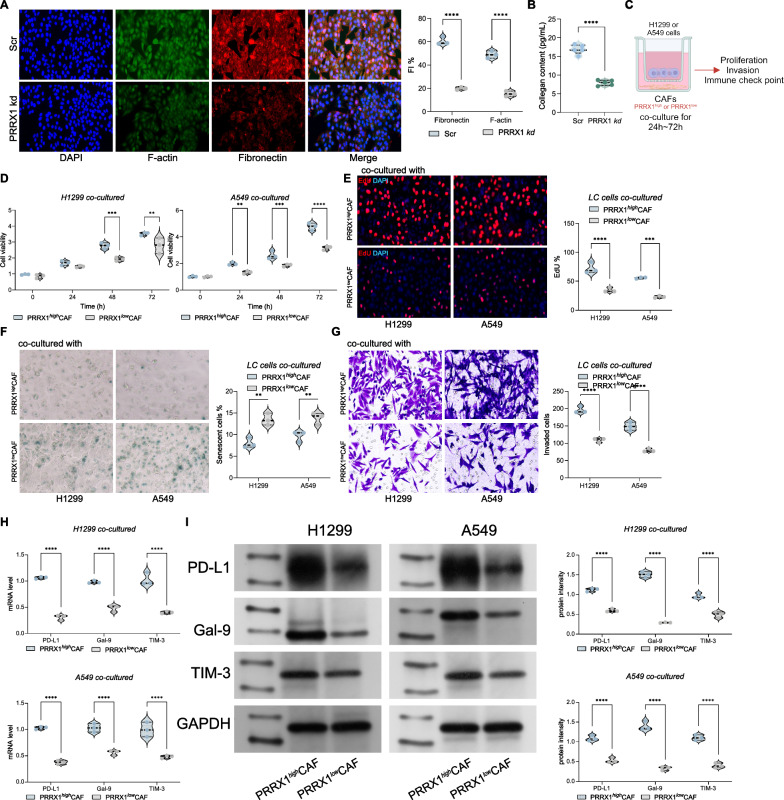
Knockdown of PRRX1 inhibits the appearance of CAF-related phenotypes and their supportive role in lung cancer cell growth. **A** Quantification of fluorescence intensity for F-actin and Fibronectin in cancer-associated fibroblasts (CAFs) via immunofluorescence detection. **B** Collagen content assay demonstrating reduced collagen production in PRRX1-knockdown CAFs compared to control. **C** Schematic representation of the co-culture system of PRRX1low/high CAFs with H1299 or A549 lung cancer cells. **D** Analysis of H1299 or A549 cell proliferation or invasion in co-culture with PRRX1low/high CAFs, indicating reduced support from PRRX1-knockdown CAFs. **E** Analysis of senescence levels in H1299 or A549 cells co-cultured with PRRX1low/high CAFs, showing reduced anti-senescence activity of PRRX1-knockdown CAFs. **F** Immunofluorescence analysis confirming reduced expression of senescence markers in H1299 or A549 cells co-cultured with PRRX1low/high CAFs. **G** Analysis of immune escape marker expression (e.g., PD-L1, Galectin-9) in H1299 or A549 cells co-cultured with PRRX1low/high CAFs, demonstrating reduced promotion by PRRX1-knockdown CAFs. **H** Western blot or qRT-PCR analysis confirming reduced expression levels of immune escape markers in H1299 or A549 cells co-cultured with PRRX1low/high CAFs. Experiments were repeated three times, and data are presented as mean ± standard deviation. Statistical analysis was performed using one-way ANOVA or 2-Way ANOVA, followed by Tukey’s post-hoc validation, **P < 0.01, ***P < 0.001

To clarify the impact of PRRX1 on CAF phenotypes and functions, we first analyzed the expression levels of Fibronectin and F-actin in PRRX1-knockdown CAFs and found a significant reduction (Fig. 6A). Additionally, the collagen content produced by these CAFs was also significantly reduced (Fig. 6B), indicating that knocking down PRRX1 can significantly weaken the CAF phenotype. Next, we further established co-culture systems of PRRX1low/high CAFs with H1299 or A549 cells (Fig. 6C). We observed that PRRX1-knockdown CAFs had a significantly reduced supportive effect on the growth and invasion of H1299 or A549 cells, as well as their anti-senescence activity (Fig. 6D–F). Furthermore, we found that PRRX1low CAFs had a significantly reduced ability to promote the expression of immune escape markers in H1299 or A549 cells (Fig. 6G, H). These results suggest that knocking down PRRX1 in CAFs can suppress the appearance of CAF-related phenotypes and their support for lung cancer cell growth, as well as their promotion of immune escape markers.

### Overexpressing OLR1 attenuates the inhibitory effect of shPRRX1 on CAFs’ functions

**Fig. 7:**
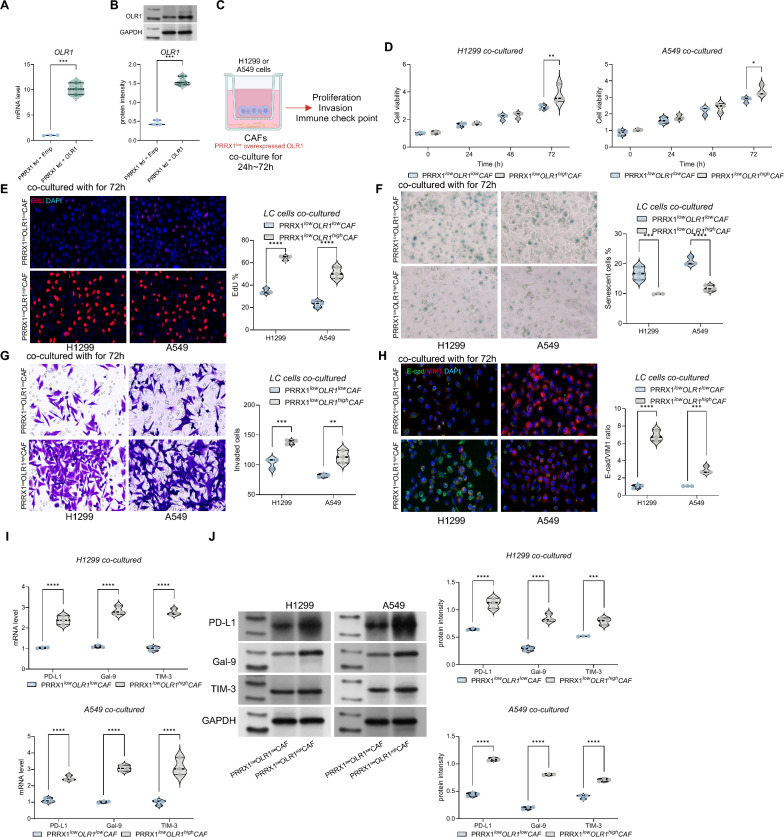
Overexpression of OLR1 attenuates the inhibitory effect of shPRRX1 on CAFs' functionality. **A**, **B** qPCR and WB analysis of OLR1 mRNA and protein expression levels in PRRX1lowCAFs after overexpression of OLR1; **C**, **D** Establishment of co-culture systems with CAFs and H1299 or A549 cells; **E** Assessment of cell viability of H1299 and A549 cells using CCK-8 assay; **F** Evaluation of DNA replication ability in H1299 and A549 cells using EdU staining; **G** Assessment of invasive capabilities of H1299 and A549 cells using Transwell assays; **H** Immunofluorescence analysis showing decreased E-cadherin and increased Vimentin (VIM1) fluorescence intensity in H1299 or A549 cells co-cultured with PRRX1-knockdown CAFs overexpressing OLR1. **I**, **J** qPCR and WB analysis of mRNA and protein expression levels of immune escape-related markers PD-L1, Galectin-9, and TIM-3 in H1299 and A549 cells. Experiments were repeated three times, and data are presented as mean ± standard deviation. Statistical analysis was performed using one-way ANOVA or 2-Way ANOVA, followed by Tukey’s post-hoc validation, **P < 0.01, ***P < 0.001

In the previous experiments, we established that both knocking down OLR1 and PRRX1 can weaken the supportive effect of CAFs on the growth and immune escape of lung cancer cells. However, whether PRRX1 can affect the function of CAFs through OLR1 requires further investigation. Therefore, we further overexpressed OLR1 in PRRX1-knockdown CAFs (Fig. 7A, B). We found that after overexpressing OLR1 in PRRX1low CAFs and co-culturing them with H1299 or A549 cells, the promoting effect on lung cancer cell growth and migration significantly increased. Additionally, it could also inhibit the senescence of lung cancer cells (Fig. 7C–G). Moreover, we found that the fluorescence intensity of E-cadherin significantly decreased, while that of Vimentin (VIM1) significantly increased in H1299 or A549 cells co-cultured with PRRX1-knockdown CAFs overexpressing OLR1 (Fig. 7H). Furthermore, the levels of PD-L1, Galectin-9, and TIM-3 expressed in H1299 and A549 cells were significantly increased (Fig. 7I, J). These results suggest that PRRX1 can influence the function of CAFs through OLR1, and overexpressing OLR1 in PRRX1-knockdown CAFs can enhance their supportive effect on lung cancer cell growth and immune escape.

## Discussion

**Fig. 8:**
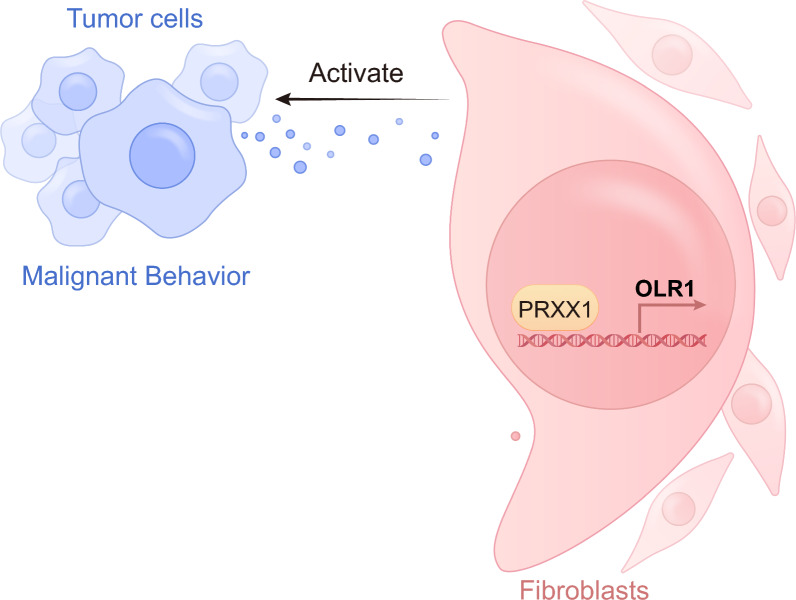
PRRX1-OLR1 as a critical modulator of CAF-mediated lung cancer progression and immune evasion

Our study illuminates the intricate web of interactions within the TME, focusing on the pivotal roles played by CAFs mediated through the PRRX1-OLR1 axis. The TME is a dynamic milieu where various cell types, including CAFs, orchestrate cancer progression. As one of the most important stromal components in the TME, CAFs have shown biological heterogeneity in many aspects, including the cell of origin, phenotype, and function [14, 15]. Understanding this heterogeneity is paramount, given its implications for cancer progression and therapeutic response (Fig. 8).

Our identification of OLR1 as significantly overexpressed in CAFs expands on existing knowledge regarding fibroblast-specific markers in cancer contexts [26]. OLR1’s association with adverse patient outcomes underscores its potential as a prognostic marker [27]. Studies have linked OLR1 overexpression with increased cancer cell proliferation, invasion, and angiogenesis, emphasizing its significance in tumor progression [28, 29]. Moreover, OLR1 has been recognized as a potential immune modulator, influencing the interactions between cancer cells and immune cells within the TME [30]. Here, we found that OLR1 was significantly overexpressed in CAFs of lung cancer and was associated with adverse patient outcomes, and OLR1^+^ CAFs promote the growth of lung cancer cells in vitro and in vivo, as well as immune evasion. Furthermore, our study delineates the regulatory relationship between PRRX1 and OLR1, adding a novel layer of complexity to the transcriptional regulation within CAFs. Transcription factors, such as PRRX1, are increasingly recognized for their roles in orchestrating the behavior of stromal cells within the TME [31–33].

The significance of our findings is underscored by the growing body of research highlighting the active participation of stromal components in cancer progression. Stromal cells, including fibroblasts, play crucial roles in tumor growth, invasion, and therapy resistance [34]. CAFs, in particular, have emerged as key regulators of these processes, emphasizing the need for targeted therapies aimed at modulating their functions [35]. Our study’s focus on the PRRX1-OLR1 axis provides a specific target for intervention, presenting a promising avenue for future therapeutic strategies.

In the context of immune evasion, our findings align with studies emphasizing the immunomodulatory roles of stromal cells. CAFs have been shown to create an immunosuppressive microenvironment, enabling cancer cells to evade immune surveillance [36–38]. Disrupting these interactions, as our study suggests by targeting the PRRX1-OLR1 axis, holds immense therapeutic potential in reactivating anti-tumor immunity.

However, it is imperative to acknowledge the limitations of our study. While our research elucidates the regulatory mechanisms involving PRRX1 and OLR1, the clinical translation of these findings demands rigorous validation. Moreover, the heterogeneity among CAF subtypes and their distinct functions in different cancer types necessitate further exploration. Additionally, the influence of other stromal components, such as pericytes and endothelial cells, on CAF behavior remains an area of active investigation. Understanding these complexities is vital for developing targeted therapies that comprehensively address the TME's diverse components.

In summary, our study elucidates the pivotal role of the PRRX1-OLR1 regulatory axis as a key determinant in the promotion of cancer-associated fibroblast (CAF)-mediated lung cancer progression and immune evasion. These findings underscore the dynamic interplay between stromal cells and tumor development, enriching our comprehension of cancer biology. While our investigation provides significant insights, ongoing endeavors in this domain remain imperative. Future research endeavors should prioritize elucidating the heterogeneity of CAFs, deciphering the complexities of interactions between stromal and immune cells, and translating these discoveries into efficacious therapeutic interventions. Only through sustained interdisciplinary investigations can we fully exploit the therapeutic potential of targeting stromal components in cancer therapy.

### Limitation

This research has made remarkable strides in understanding the intricate mechanisms underlying cancer progression and immune evasion, particularly through the elucidation of the PRRX1-OLR1 regulatory axis in cancer-associated fibroblasts (CAFs). However, as with any study, there are inherent limitations and avenues for future exploration. One significant limitation is the complexity and heterogeneity of CAF populations. CAFs exhibit diverse phenotypes and functions based on their origin, localization within the tumor microenvironment, and interactions with other cell types. Future research should delve deeper into characterizing the different subtypes of CAFs and understanding their distinct roles in tumor progression. This research sheds light on the interplay between stromal cells and cancer progression, but further investigation into the complexities of interactions between stromal and immune cells is warranted. The tumor microenvironment is a dynamic ecosystem where various cell types communicate and influence each other. Understanding these interactions is crucial for developing effective immunotherapeutic strategies. Overall, this study provides a solid foundation for further exploration into the role of stromal components in lung cancer treatment.

### Supplementary Information

Supplementary Material 1.

## Data Availability

Data will be made available on request.
