# Supplementary material for: PRRX1-OLR1 axis supports CAFs-mediated lung cancer progression and immune suppression
Source: Cancer Cell Int. 2024 Jul 15;24:247. doi: 10.1186/s12935-024-03436-9 (PMC11251326; doi:10.1186/s12935-024-03436-9)
Supplement: Supplementary file 1 — Supplementary Material 1. [file 12935_2024_3436_MOESM1_ESM.docx]

| **Gene Symbol** | **logFC** | **t** | **adj.P.Val (FDR)** |
| --- | --- | --- | --- |
| IGFBP5 | 4.0851012 | 3.798443907 | 0.071308672 |
| GZMD | 3.598753 | 3.714741545 | 0.073945722 |
| ASB5 | 3.4994642 | 4.543229673 | 0.050795126 |
| IL1A | 3.4600094 | 5.57133058 | 0.035449264 |
| SOX11 | 3.284768 | 6.721334708 | 0.028335376 |
| IL11 | 3.2512438 | 4.307767981 | 0.055422832 |
| GZME | 3.2463136 | 4.120997096 | 0.060305911 |
| MCPT8 | 3.1161478 | 4.273472561 | 0.055929435 |
| PRND | 3.1061946 | 8.100720579 | 0.018184366 |
| CSF3 | 2.9432946 | 9.230089624 | 0.013859546 |
| SORBS2 | 2.8918634 | 6.305465938 | 0.028335376 |
| KCNK10 | 2.7720588 | 5.5989541 | 0.035230229 |
| OBFC2A | 2.7465046 | 6.731440679 | 0.028335376 |
| SLC26A7 | 2.742293 | 4.938291863 | 0.04252213 |
| RGS16 | 2.729292 | 6.309907702 | 0.028335376 |
| SEPTIN4 | 2.7266948 | 6.986836023 | 0.027058214 |
| GM2115 | 2.69737 | 3.709996765 | 0.074057511 |
| NR4A2 | 2.6713054 | 5.065082955 | 0.040432137 |
| PLA1A | 2.6108806 | 4.075637942 | 0.06176188 |
| GALNTL4 | 2.5594548 | 4.295380099 | 0.055474765 |
| DTNA | 2.5286086 | 10.07902379 | 0.013859546 |
| DKK2 | 2.4410322 | 6.216554482 | 0.028335376 |
| OLR1 | 2.3664836 | 7.150028254 | 0.024592642 |
| LIF | 2.3540222 | 6.338648012 | 0.028335376 |
| S100B | 2.3099836 | 5.270153925 | 0.038043139 |
| SPRR2H | 2.302844 | 5.525206866 | 0.035573973 |
| PTGES | 2.2853008 | 4.624389768 | 0.048989515 |
| CD24A | 2.2199636 | 5.426915244 | 0.036004127 |
| SYT7 | 2.2189862 | 5.312831997 | 0.037588354 |
| SYNPO2 | 2.2061122 | 4.598071128 | 0.049590006 |
| NIPAL1 | 2.1751012 | 4.522631196 | 0.051460289 |
| PTGS2 | 2.138392 | 4.129213333 | 0.060010637 |
| ABCB1A | 2.1338214 | 7.216836081 | 0.024592642 |
| BDKRB2 | 2.1309202 | 4.204768992 | 0.05787814 |
| KCNQ5 | 2.09582 | 5.253785826 | 0.038173939 |
| MFAP3L | 2.0935112 | 5.55922756 | 0.035573973 |
| SYTL2 | 2.0782056 | 5.796624723 | 0.034587885 |
| SLCO4A1 | 2.0737856 | 3.683443986 | 0.075599342 |
| KRT20 | 2.0733366 | 6.515099811 | 0.028335376 |
| KCNMB1 | 2.0596732 | 4.325026303 | 0.055120206 |
| QPCT | 1.9091326 | 4.376698867 | 0.054150974 |
| GDNF | 1.901106 | 3.820665535 | 0.070462381 |
| EREG | 1.894778 | 5.334376511 | 0.037157931 |
| LRRC8D | 1.8731454 | 8.846479434 | 0.013859546 |
| UCHL1 | 1.857248 | 6.738068868 | 0.028335376 |
| IL6 | 1.823254 | 3.80517893 | 0.071007922 |
| CCRN4L | 1.7936676 | 4.161756049 | 0.058948708 |
| FEZ1 | 1.7791776 | 4.03502476 | 0.06236781 |
| TMCC3 | 1.7441296 | 4.900810114 | 0.04342725 |
| GNG2 | 1.743669 | 5.251551218 | 0.038173939 |
| THSD7A | 1.7431372 | 7.067730785 | 0.026095793 |
| VGF | 1.7428556 | 3.998382598 | 0.06403744 |
| CALB2 | 1.71812 | 4.189192953 | 0.058004795 |
| HAVCR2 | 1.7084342 | 4.422972043 | 0.053067653 |
| SLC6A17 | 1.706366 | 9.110773259 | 0.013859546 |
| CCL7 | 1.685798 | 3.739145374 | 0.073204475 |
| NGF | 1.6823768 | 3.712452656 | 0.074041137 |
| FBXO32 | 1.6662688 | 3.936781118 | 0.066562066 |
| NT5E | 1.6541518 | 4.296564029 | 0.055474765 |
| DUSP10 | 1.6411158 | 3.805281437 | 0.071007922 |
| SLC2A13 | 1.6369908 | 3.768784005 | 0.072437409 |
| NRCAM | 1.6335324 | 3.731865341 | 0.073465941 |
| 2310005G13RIK | 1.6136932 | 4.0458203 | 0.061991328 |
| SGMS2 | 1.6134534 | 6.457310733 | 0.028335376 |
| ODC1 | 1.6101234 | 4.442941822 | 0.053048797 |
| CCL2 | 1.605868 | 3.931564917 | 0.066809652 |
| PITPNC1 | 1.592187 | 10.31574587 | 0.013859546 |
| LOC100044234 | 1.57904 | 4.118745607 | 0.060305911 |
| RASSF10 | 1.5389238 | 5.12414914 | 0.040130556 |
| PDE7B | 1.5302954 | 6.313405692 | 0.028335376 |
| CREM | 1.5037284 | 8.223353091 | 0.018184366 |
| 1810011O10RIK | 1.494148 | 4.75857006 | 0.045378134 |
| ACSL6 | 1.494018 | 4.887351291 | 0.04342725 |
| PTCH1 | 1.4830198 | 4.157614327 | 0.059176414 |
| SYT13 | 1.4559204 | 4.784956491 | 0.045201961 |
| THBD | 1.452148 | 4.092988923 | 0.061472986 |
| SEMA7A | 1.4506488 | 4.173513594 | 0.058421491 |
| TNFRSF9 | 1.4461242 | 4.105083804 | 0.060907261 |
| EPHA4 | 1.445067 | 6.461345856 | 0.028335376 |
| SLCO3A1 | 1.428906 | 3.770632757 | 0.072346523 |
| NPTX2 | 1.4277948 | 4.046169493 | 0.061991328 |
| LRRK2 | 1.427063 | 5.246817502 | 0.038173939 |
| B130021B11RIK | 1.424891 | 3.729518152 | 0.073468279 |
| SLC20A1 | 1.424182 | 4.934701485 | 0.04252213 |
| CFLAR | 1.409141 | 6.40203097 | 0.028335376 |
| LIPH | 1.4056714 | 4.18343923 | 0.05812991 |
| ENAH | 1.4048064 | 5.216097581 | 0.038900557 |
| DENR | 1.4006616 | 3.948773527 | 0.066306085 |
| LOC640502 /// UAP1 | 1.398694 | 5.559120061 | 0.035573973 |
| ZDHHC14 | 1.3898944 | 4.518093167 | 0.051599733 |
| PRSS46 | 1.3865512 | 8.644042644 | 0.013859546 |
| TTPAL | 1.3862566 | 5.909007289 | 0.034103692 |
| MLLT11 | 1.3712862 | 6.737788689 | 0.028335376 |
| HECTD2 | 1.3588504 | 6.55731693 | 0.028335376 |
| CXCL14 | 1.348044 | 3.756139525 | 0.072750679 |
| HIPK2 | 1.3338786 | 3.977564577 | 0.06516082 |
| TUBA8 | 1.3195572 | 4.087346874 | 0.061480208 |
| KCNJ8 | 1.3192528 | 4.745155782 | 0.045547467 |
| AKAP2 | 1.276458 | 5.434470913 | 0.036004127 |
| A330021E22RIK | 1.2679562 | 4.553551693 | 0.050356804 |
| IER3 | 1.264566 | 5.277890005 | 0.038043139 |
| GFOD1 | 1.2625818 | 5.79242669 | 0.034587885 |
| NFKBIZ | 1.260883 | 3.911379053 | 0.067355575 |
| ASB2 | 1.236552 | 4.299933778 | 0.055474765 |
| AI848100 | 1.2294662 | 5.45186509 | 0.035954485 |
| SLC22A4 | 1.2261494 | 4.292404769 | 0.055474765 |
| CDKL2 | 1.2228992 | 4.899151445 | 0.04342725 |
| TNFAIP3 | 1.2158192 | 5.003220374 | 0.041387468 |
| LOC100045317 | 1.184873 | 3.72778503 | 0.073468279 |
| ARHGAP6 | 1.1838992 | 4.317552174 | 0.055120206 |
| GOLGA4 | 1.1838226 | 4.565939753 | 0.050102741 |
| CLCF1 | 1.1803294 | 5.940862869 | 0.034103692 |
| CD44 | 1.180178 | 3.732029627 | 0.073465941 |
| TNFRSF23 | 1.1770044 | 4.11258955 | 0.060477334 |
| CELF4 | 1.175372 | 5.058954687 | 0.04045195 |
| 1700047I17RIK1 | 1.169003 | 3.910081469 | 0.067355575 |
| ZC3H12A | 1.1680428 | 5.413206171 | 0.036004127 |
| NFKBIA | 1.1676 | 4.245397627 | 0.056736054 |
| AP1S2 | 1.1664864 | 5.275067861 | 0.038043139 |
| PLEKHG1 | 1.155623 | 4.058549827 | 0.061991328 |
| TIAM2 | 1.1527024 | 7.711396738 | 0.021940733 |
| NR4A3 | 1.1521628 | 5.067652616 | 0.040432137 |
| GCNT2 | 1.1457948 | 6.632191918 | 0.028335376 |
| WFS1 | 1.1408656 | 4.031892293 | 0.062421702 |
| SIPA1L3 | 1.1407376 | 4.993608307 | 0.041703599 |
| ABCC4 | 1.1399334 | 6.875940946 | 0.028335376 |
| CCND2 | 1.1341788 | 4.70906926 | 0.046575961 |
| P2RY2 | 1.1311296 | 4.17715188 | 0.058243324 |
| MTAP1B | 1.1285328 | 5.006136493 | 0.041387468 |
| LPAR4 | 1.1278088 | 5.809740695 | 0.034587885 |
| RNF125 | 1.1216548 | 3.889677784 | 0.06847248 |
| RABGAP1L | 1.1204944 | 5.034422168 | 0.04091327 |
| VCAM1 | 1.1124014 | 3.825710256 | 0.070121823 |
| CPD | 1.112017 | 3.993192812 | 0.064125075 |
| MAPRE2 | 1.1096564 | 6.672473072 | 0.028335376 |
| 1110012J17RIK | 1.106141 | 5.079476366 | 0.040396684 |
| TNFRSF22 | 1.1043028 | 3.88505368 | 0.068566403 |
| ARMCX4 | 1.1001216 | 3.829690799 | 0.070121823 |
| GSTO2 | 1.0996334 | 4.861534391 | 0.043602342 |
| FYN | 1.0995486 | 4.026862734 | 0.062530035 |
| FAM82A1 | 1.095541 | 6.624486077 | 0.028335376 |
| SH3KBP1 | 1.0906458 | 4.751055877 | 0.045547467 |
| DIXDC1 | 1.0856512 | 6.326380268 | 0.028335376 |
| AKAP9 | 1.0845666 | 8.671620786 | 0.013859546 |
| STK38L | 1.076918 | 8.578823054 | 0.013859546 |
| APBB2 | 1.0726926 | 4.833606214 | 0.044674358 |
| PRKX | 1.065537 | 4.006700387 | 0.063633918 |
| AFF2 | 1.0650416 | 4.370175053 | 0.054571114 |
| SLC12A2 | 1.0604378 | 5.140682468 | 0.040130556 |
| GSTO1 | 1.060226 | 3.727828899 | 0.073468279 |
| TRPV2 | 1.0557332 | 4.573090983 | 0.049906338 |
| ITPRIPL2 | 1.044892 | 4.24814545 | 0.056576043 |
| ABCA1 | 1.0443234 | 3.764911551 | 0.0726736 |
| TNFRSF22 | 1.0434666 | 4.057270646 | 0.061991328 |
| TJP2 | 1.0341586 | 3.697647593 | 0.074696832 |
| ADORA1 | 1.0333544 | 4.771426945 | 0.045201961 |
| RASL12 | 1.0327284 | 3.761788892 | 0.072681934 |
| EPM2AIP1 | 1.0300424 | 7.145013342 | 0.024592642 |
| BC005512 | 1.0284072 | 3.807876839 | 0.071007922 |
| TTC39B | 1.0210582 | 3.716582889 | 0.073855423 |
| INTS6 | 1.0164158 | 4.416457631 | 0.053307146 |
| TXNDC9 | 1.0149284 | 5.908947824 | 0.034103692 |
| ACSS2 | 1.0021828 | 4.648934433 | 0.048047635 |
| SH3BGR | 1.0001564 | 3.743573275 | 0.073174134 |
| SLC43A3 | -1.319762 | -5.539661842 | 0.035573973 |
| CDKN1B | -1.3155244 | -5.492257395 | 0.035573973 |
| 5033421C21RIK | -1.1201258 | -5.343744602 | 0.037125545 |
| NFIB | -1.0083228 | -5.134728868 | 0.040130556 |
| STARD10 | -1.1873538 | -4.474280466 | 0.052841317 |
| FAM132A | -1.001401 | -4.427269738 | 0.053067653 |
| IGF1 | -1.0038622 | -4.306670483 | 0.055430988 |
| ANXA8 | -1.7253 | -3.942140857 | 0.066518815 |
| TGTP1 | -1.7828062 | -3.80929464 | 0.071007922 |
| BICC1 | -1.262062 | -3.763566824 | 0.0726736 |
| RASL11A | -1.104772 | -3.733644068 | 0.073436515 |
